# Laparoscopic cholecystectomy after conservative subcapsular hepatic hematoma management: A rare case report

**DOI:** 10.1016/j.ijscr.2023.109162

**Published:** 2023-12-15

**Authors:** Anung Noto Nugroho, Ida Bagus Budhi Surya Adnyana, Kristanto Yuli Yarso, Monica Bellynda, Riza Setya Agrensa, Faizal Muhammad

**Affiliations:** a Digestive Division of Surgery Department, Faculty of Medicine, Sebelas Maret University, Surakarta 57126, Indonesia; b Oncology Division of Surgery Department, Faculty of Medicine, Sebelas Maret University, Surakarta 57126, Indonesia; c General Surgery Department, Faculty of Medicine, Sebelas Maret University, Surakarta 57126, Indonesia

**Keywords:** Endoscopic retrograde cholangiopancreatography, Jaundice, Subcapsular hepatic hematoma, Liver, Abdominal pain

## Abstract

**Introduction and importance:**

Endoscopic Retrograde Cholangiopancreatography (ERCP) is a less invasive procedure to diagnose and treat biliary disease. However, it has a mortality rate of 0.43–1 %. ERCP has several complication that can arise, one of which is a subcapsular hepatic hematoma (SCH). Incidence of subcapsular hematoma is about 1 %.

**Case presentation:**

In this case we reported a 33-years-old female complained of jaundice in the entire and right upper abdominal pain. She underwent ERCP and stent placement due to an obstruction in the biliary system. The day after ERCP, she has complained about persistent sharp pain on the upper abdomen. Abdominal ultrasound showed SCH. She then underwent laparoscopic diagnostic and showed the hematoma at the subcapsular of the right upper lobe.

**Clinical discussion:**

Then it was decided to conservative therapy with an antibiotic and analgesics. Cholecystectomy was also performed to treat cholelithiasis. Patient discharge from hospital in three days after surgery with a good condition and no symptom about stomachache.

**Conclusion:**

Conservative treatment is the goal while managing SCH in a good hemodynamic state. Once a hematoma has been identified, treatment with a broad-spectrum antibiotic should be started since the hematoma may turn into a secondary infection that requires invasive techniques and drainage.

## Highlights

• Endoscopic Retrograde Cholangiopancreatography (ERCP) is a less invasive procedure to diagnose and treat biliary disease.
• ERCP has a mortality rate of 0.43-1%. ERCP has several complication that can arise, one of which is a subcapsular hepatic hematoma (SCH).
• SCH is a rare complication of ERCP resulting from guide wire damage to intrahepatic vessels.
• Pancreatitis, cholangitis, perforation, and bleeding due to papillotomy are the foremost common causes of SCH.
• This rare condition has an incidence rate around 2.5% to 8% and the mortality rate was 0.5% -1%.

## 1 Introduction

Endoscopic retrograde cholangiopancreatography (ERCP) is a less invasive procedure to diagnose and treat pancreatic-gall disease (pancreatitis and cholangitis). ERCP is more effective and efficient than open procedure. It is very useful because it can reach the area that cannot be reached out by open procedure. The patient should at general anesthesia during ERCP procedure. Moreover, this procedure is easy and cost-effective as it is usually covered by health insurance [1].

Subcapsular hepatic hematoma (SCH) is a rare complication of ERCP resulting from guide wire damage to intrahepatic vessels. Pancreatitis, cholangitis, perforation, and bleeding due to papillotomy are the foremost common causes of SCH. This rare condition has an incidence rate around 2.5 % to 8 % and the mortality rate was 0.5 %–1 %. However, the high morbidity rate can be seen from length of stay at the hospital as well [2]. This case has been written according to latest Surgical CAse REport (SCARE) Guidelines [3].

## 2 Presentation of case

**Fig. 1:**
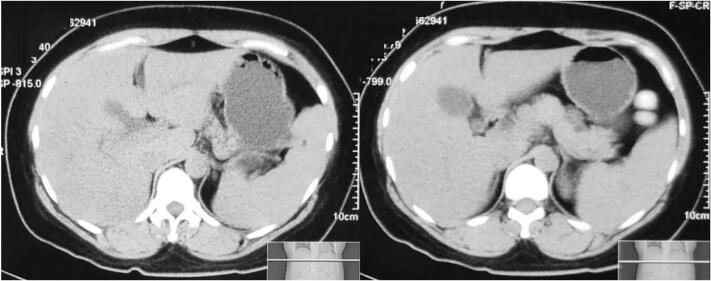
Pre-operative CT-scan showed cholelithiasis and cholecystitis as well in the left lobe intrahepatic biliary duct dilatation, and cystic duct dilatation.

A 33-years-old woman complained of jaundice at the whole body and right upper abdominal pain since 1 month ago, without fever. The physical examination showed jaundice from sclera to the whole body. An abdominal CT-scan examination was perform cholelithiasis found accompanied by a dilatation of the both lobe intrahepatic biliary duct and dilated cystic duct (Fig. 1). Based on laboratory tests, there was an increase at AST 256, ALT 388, and bilirubin total 6.34 mg/dL, indirect bilirubin 0.7 mg/dL, and direct bilirubin 5.23 mg/dL, normal result for Clotting Time/Bleeding Time, and normal hemoglobin decrease (Hb 11.5 mg/dL). Patient was planned to have ERCP procedure for diagnostic and treatment and conduct general surgery preparation.

**Fig. 2:**
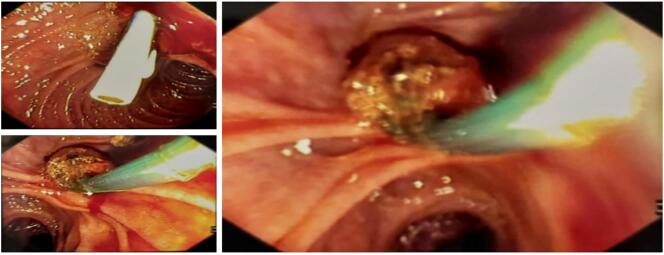
The endoscopic retrograde cholangiopancreatography procedure found common bile duct obstruction and performed plastic stent placement.

**Fig. 3:**
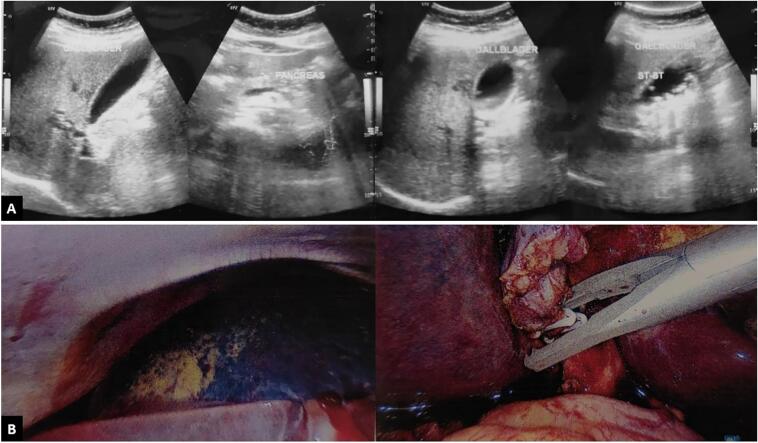
(A) The ultrasound following ERCP showed hepatomegaly with a hematoma in the hepatic right lobe; (B) laparoscopic procedure showed 6 × 4 × 1 cm3 hematoma of hepatic subcapsular right lobe.

The patient underwent an ERCP procedure in general anesthesia. Trough mouth opening guide wire was placed and insert to duodenum and go deeper to Ampulla of Vater under continuous C-Arm surveillance. During the procedure, we found an obstruction at the common bile duct (CBD) caused by single stone size 10 × 8 mm. the stones was successfully extracted using dormia basket and plastic stent placed on CBD (Fig. 2). During the procedure, the right intrahepatic duct was suddenly narrowed. It showed there was compression from outer wall. We decided to stop the procedure and perform abdominal ultrasound (Fig. 3A), it showed right lobe hepatomegaly with a hematoma in the right lobe with size 6 × 4 cm. Hemoglobin was dropped into 7.1 mg/dL following this hematoma. Furthermore, the AST and ALT were 231 and 295, respectively.

The patient sent to ward with an observation for the right hepatic hematoma. The clinical sign were pain. The patient gets an adequate antibiotic of Ampicillin-Sulbactam 50 mg/kg/12 h and Amoxicillin 45 mg/kg/12 h. Painkiller was administered as well. The complaint was well managed by those medications. After 36 h of observation, the clinical sign of this patient is getting better and underwent laparoscopic diagnostic procedure and showed the hematoma at the subcapsular of the right lobe of the liver about 7 × 5 × 0.5 cm^3^ without pulsating (Fig. 3B). During the procedure, the bleeding was evaluated for 10–15 min and we didn't find any hematoma expansion as well as pulsation. After no active bleeding was confirmed we decided to continue the procedure with retrograde cholecystectomy, artery and cystic duct identified was clipped independently. Surgery was done without any complication. Patient discharged from hospital in three days after surgery with adequate painkiller without any symptoms.

## 3 Clinical discussion

SCH may be uncommon difficulty of ERCP resulting from guide wire damage to intrahepatic vessels [4]. The clinical signs are frequently as single abdominal pain coexisting with anemia or fever. This pain usually happens within 24 h after ERCP. Ultrasound, CT-scan, or MRI are diagnostic modalities that may precisely investigate the location and size SCH. Moreover, laboratory studies are required to recognize acute anemia that may result from a hematoma [2].

Bleeding complications variety from slight mucosal injury and submucosal bleeding to symptomatic blood loss due to organ damage requiring numerous transfusions [5]. Patients utilizing antiplatelet, anticoagulants and with coagulopathy have a better change of bleeding, be that as it may, with particular procedural methods, mid-ERCP also can increment the chance of bleeding, counting zipping, sphincterotomy, and utilizing of a needle-blade sphincterotome. Bleeding may take place because of papillary manipulation and guide wire brought on damage. The hypothesized mechanism of damage that causes hematoma includes perforation of the intrahepatic biliary tree wall in the course of the development of the device (i.e., guide wires), coming about in demanding rupture of small intrahepatic vessels. The blood at that point enters through the liver parenchyma to make a hematoma, a collection of blood contained within liver capsule [6].

The pathophysiological mechanisms of these hematomas are not completely caught on, and a few theories had been proposed. A hypothesis proposes that amid biliary swelling, tractional weight develops, inflicting little biliary vessels to rupture, coming about in intraparenchymal hemorrhage and subcapsular pooling because of centrifugal bloodstream. Agreeing with another hypothesis, the guide wire utilized in ERCP can purpose damage to the little diameter vessels in the bile duct, coming about in damage and bleeding [2,7]. Thus, complications of interventional radiology are encountered more often due to the rise in use of interventional radiology to the extent that lines between specialties are becoming blurred as radiologists are doing more and more invasive procedure.

This mechanism moreover legitimizes the nearness of discussion within the hematoma. In any case, SCH was reported when CBD stones were extricated without utilizing direct guide wires. In addition, there are moreover a few distributed reviews of unexplained hematoma after laparoscopic cholecystectomy. Anatomical and hemodynamic traits of this locale, which includes bad subdiaphragmatic stress and “third flow” of blood from different resources, additionally play a function. At last, the nearness of unusual ectatic vessels at the liver floor, as discovered in our case, may propose a part for few vascular malformations [8]. Thus, the most often hypothesized process involves neovascularization, fibrin deposition, hepatic sinusoidal blockage, endothelial damage owing to preeclampsia, and microhemorrhage resulting in hematoma development.

Management of SCH in good hemodynamic status is conservative treatment. Once distinguished with hematoma ought to be begun on broad-spectrum anti-microbial since the hematoma can become a secondary infection, coming about in sepsis and the capability requirement for drainage and invasive methods. This SCH ought to be suspicious in patients who develop clinical highlights such as stomachache, paleness after ERCP, within laboratory discoveries appearing diminished hemoglobin and hematocrit levels [7]. However, if the patient's hemodynamic is deteriorated indicating the bleeding is actively growing, a drainage or laparoscopy with irrigation can be considered. Furthermore, Artificial intelligence and autonomous actions in surgery can be considered as well despite challenges of deep learning is remaining [9].

## 4 Conclusion

One of the rare complications of ERCP is SCH. In this case, a 32 year old female who has had ERCP to treat her cholelithiasis. She then developed signs and symptoms indicating SCH happens within 24 h after ERCP. Management of SCH depends on the patient condition. In this case, we used conservative therapy with evaluation during the laparoscopic and with analgesics, antibiotic therapy.

## Source of support

The authors are grateful to all acupuncture and neurology colleagues in Faculty of Medicine, Sebelas Maret University.

## Ethical approval

This case study was approved by Health Research Ethics Committee of Sebelas Maret University, No. 131/UN27.06.11/KEP/EC/2023. Written informed consent was obtained from the patient for publication of this case report and accompanying images. A copy of the written consent is available for review by the Editor-in-Chief of this journal on request.

## Financial disclosure

This case study did not receive any specific grant from funding agencies in the public, commercial, or not-for-profit sectors.

## CRediT authorship contribution statement

Anung Noto Nugroho, Ida Bagus Budhi Surya Adnyana, Kristanto Yuli Yarso: conceptualization, surgical intervention, treatment, research materials, logistic support, writing-reviewing, supervision and validation. Monica Bellynda, Riza Setya Agrensa, Faizal Muhammad: treatment evaluation, medical follow-up, writing-original initial draft and final manuscript.

## Guarantor

Kristanto Yuli Yarso.

## Research registration

N/A.

## Declaration of competing interest

The authors declare that they have no competing interests.

## References

[1] Sanders D.J., Bomman S., Krishnamoorthi R., Kozarek R.A. (2021). Endoscopic retrograde cholangiopancreatography: current practice and future research, world. J. Gastrointest. Endosc..

[2] Zappa M.A., Aiolfi A., Antonini I., Musolino C.D., Porta A. (2016). Subcapsular hepatic haematoma of the right lobe following endoscopic retrograde cholangiopancreatography: case report and literature review. World J. Gastroenterol..

[3] Agha R.A., Franchi T., Sohrabi C., Mathew G., Kerwan A., Thoma A., Beamish A.J., Noureldin A., Rao A., Vasudevan B., Challacombe B., Perakath B., Kirshtein B., Ekser B., Pramesh C.S., Laskin D.M., Machado-Aranda D., Miguel D., Pagano D., Millham F.H., Roy G., Kadioglu H., Nixon I.J., Mukherjee I., McCaul J.A., Chi-Yong Ngu J., Albrecht J., Rivas J.G., Raveendran K., Derbyshire L., Ather M.H., Thorat M.A., Valmasoni M., Bashashati M., Chalkoo M., Teo N.Z., Raison N., Muensterer O.J., Bradley P.J., Goel P., Pai P.S., Afifi R.Y., Rosin R.D., Coppola R., Klappenbach R., Wynn R., De Wilde R.L., Surani S., Giordano S., Massarut S., Raja S.G., Basu S., Enam S.A., Manning T.G., Cross T., Karanth V.K., Kasivisvanathan V., Mei Z., The S.C.A.R.E. (2020). Guideline: updating Consensus Surgical CAse REport (SCARE) guidelines. Int. J. Surg..

[4] Pivetta L.G.A., da Costa Ferreira C.P., de Carvalho J.P.V., Konichi R.Y.L., Kawamoto V.K.F., Assef J.C., Ribeiro M.A. (2020). Hepatic subcapsular hematoma post-ERCP: case report and literature review. Int. J. Surg. Case Rep..

[5] Taher H., Kidr E., Kamal A., ElGobashy M., Mashhour S., Nassef A., Tawfik S., El Tagy G., Shaban M., Eltantawi H., Abdullateef K.S. (2023). Transhepatic ultrasound guided embolization as a successful novel technique in treatment of pediatric complex intrahepatic arterioportal fistula: a case report and review of the literature. J Med Case Reports.

[6] Singh P., Warren K., Collier V. (2020). Ruptured subcapsular liver hematoma: a rare complication of HELLP syndrome. Case Reports Hepatol..

[7] Grigorakis S., Tzimas G.N., Alexakis C., Morea B.E., Kontomitros N. (2022). Subcapsular liver hematoma: a rare complication of hemolysis, elevated liver enzymes, and low platelets (HELLP) syndrome managed conservatively. Cureus.

[8] Petrucci R., Das A. (2021). Subcapsular hepatic hematoma post-endoscopic retrograde cholangiopancreatography requiring surgical necrosectomy. J. Med. Cases..

[9] Taher H., Grasso V., Tawfik S., Gumbs A. (2022). The challenges of deep learning in artificial intelligence and autonomous actions in surgery: a literature review. Artif. Intell. Surg..

